# Supplementary material for: Scoping review of evidence synthesis: Concepts, types and methods
Source: PLoS One. 2025 May 16;20(5):e0323555. doi: 10.1371/journal.pone.0323555 (PMC12084050; doi:10.1371/journal.pone.0323555)
Supplement: S1 Appendix — (DOCX) [file pone.0323555.s001.docx]

**S1 Appendix Search strategies used for each database**

| **Database** | **Search strategies** | **Results** |
| --- | --- | --- |
| PUBMED | (("Systematic Reviews as Topic"[MeSH Terms] OR "MetaAnalysis as Topic"[MeSH Terms] OR "Review Literature as Topic"[Mesh]) OR ("Systematic Review"[Title] OR "MetaAnalysis"[Title] OR "Scoping review"[Title] OR "Narrative  review"[Title] OR "Systematic qualitative review"[Title] OR  "Systematic evidence review"[Title] OR "Systematic quantitative review"[Title] OR "Qualitative systematic review"[Title] OR "Systematic meta-review"[Title] OR "Systematic critical review"[Title] OR "Systematic mixed"[Title] OR "Mixed methods review"[Title] OR "Cochrane review"[Title] OR "Integrative review"[Title] OR "Literature review"[Title] OR "Umbrella review"[Title] OR "Rapid review"[Title] OR "Meta-synthesis"[Title] OR Overview[Title] OR "Realist review"[Title] OR "Critical review"[Title] OR "Mapping review"[Title] OR "Evidence map*"[Title] OR Summary[Title] OR "Systematized review"[Title] OR "Evidence synthesis"[Title] OR "Qualitative evidence synthesis"[Title] OR "Policy brief"[Title] OR "Evidence brief"[Title] OR "Living systematic review"[Title] )) AND (Typolog*[Title/Abstract] OR Methodology[Title/Abstract]) | 20,974 |
| Embase | #1 'systematic review (topic)'/exp AND  [embase]/lim  #2 'meta analysis (topic)'/exp AND  [embase]/lim  #3 ('systematic review':ti OR 'meta-analysis':ti  OR 'scoping review':ti OR 'narrative review':ti  OR 'systematic qualitative review':ti OR  'systematic evidence review':ti OR 'systematic  quantitative review':ti OR 'qualitative  systematic review':ti OR 'systematic metareview':ti OR 'systematic critical review':ti OR  'systematic mixed':ti OR 'mixed methods  review':ti OR 'cochrane review':ti OR  'integrative review':ti OR 'literature review':ti  OR 'umbrella review':ti OR 'rapid review':ti OR  'meta-synthesis':ti OR overview:ti OR 'realist  review':ti OR 'critical review':ti OR 'mapping  review':ti OR 'evidence map':ti OR summary:ti  OR 'systematized review':ti OR 'evidence  synthesis':ti OR 'qualitative evidence  synthesis':ti OR 'policy brief':ti) AND  [embase]/lim  #4 (typolog*:ab,ti OR methodology:ab,ti) AND  [embase]/lim  #5 #1 OR #2 OR #3  #6 #5 AND #4 | 19,147 |
| BVS | mh:("Systematic Reviews as Subject" OR "Systematic  Reviews as Topic" OR "Revisiones Sistemáticas como Asunto") OR mh:("Review Literature as Topic" OR "Literatura de Revisión como Asunto") OR mh:("Meta-Analysis as Topic" OR "Metaanálisis como Asunto") OR ti:("Systematic Review" OR "Meta-Analysis" OR "Scoping review" OR "Narrative review" OR "Systematic qualitative review" OR "Systematic evidence review" OR "Systematic quantitative review" OR "Qualitative systematic review" OR "Systematic meta-review" OR "Systematic critical review" OR "Systematic mixed" OR "Mixed methods review" OR "Cochrane review" OR "Integrative review" OR "Literature review" OR "Umbrella review" OR "Rapid review" OR "Meta-synthesis" OR overview OR "Realist review" OR "Critical review" OR "Mapping review" OR "Evidence map" OR  summary OR "Systematized review" OR "Evidence synthesis" OR "Qualitative evidence synthesis" OR "Policy brief") AND  ti:((typolog* OR methodology) OR ab:(typolog* OR methodology)) AND ( db:("LILACS" OR "BDENF" OR "IBECS" OR "BBO" OR "INDEXPSI" OR "PREPRINT-MEDRXIV" OR "colecionaSUS" OR "BRISA")) | 1,422 |
| Total with  duplicates |  | 41,543 |
| Total |  | 28,045 |

**Access date: Nov/13/2024**
