# Supplementary material for: Scoping review of evidence synthesis: Concepts, types and methods
Source: PLoS One. 2025 May 16;20(5):e0323555. doi: 10.1371/journal.pone.0323555 (PMC12084050; doi:10.1371/journal.pone.0323555)
Supplement: S2 Appendix — (DOCX) [file pone.0323555.s002.docx]

**S2 Appendix Characteristics for each method of evidence synthesis.**

| **Name** | **Characteristics** | **Situation in which it should be used** | **Stages** | **Data extraction and analysis** | **Available guidelines for using synthesis and/or examples of synthesis** |
| --- | --- | --- | --- | --- | --- |
| 1. Inventory of references | It consists of identifying and listing bibliographical references and other data [7,8]. | To demonstrate what evidence is available, to confirm the necessity for an additional review, and when evidence is required quickly to support an action. Execution time: from 3 days to 02 months [7,8]. | 1. Definition of the research question.  2. Definition of methods and shortcuts (question scope limitation, date limitation)  3. Research in databases.  4. Screening of titles and abstracts.  5. Data extraction from the abstracts of the included studies.  6. Results report [7,8]. | It only presents the results, such as endpoints and findings, which were detailed in the abstracts [7,8]. | **Example of a study:** List of references on international recommendations for autopsies in the event of COVID-19 deaths  <https://docs.bvsalud.org/biblioref/2020/04/1087821/3-inventarioreferenciarecomendacaointernacionalnecropsiacovid1_cAkBxur.pdf> |
| 2. Rapid review | Evidence synthesis with systematized, simplified steps to produce information in less time and with fewer resources to support decision-making. [1,7–18 ,18,20,21] | For decision-making in health policy and practice, when there is a need for evidence in a short period of time, using methodological shortcuts such as: limitations on the scope of the research question, language limits, among others. The runtime ranges from 3 days to 6 months [1,8–18 ,18,20,21]. | 1. Team formation  2. Definition of the research question  3. Definition of methods and methodological shortcuts  4. Development of inclusion criteria  5. Definition the research strategy  6. Screening stage for titles and abstracts  7. Reports sought for retrieval  8. Screening and reading of the full article  9. Data extraction  10. Assessing the quality of evidence  11. Report of the results [9,11,12,16-18 ,20,21]. | Depending on the methodological shortcuts defined, it may have more simplified extraction and analysis, with or without assessment of the methodological quality of the studies, performed by one reviewer and checked by another [9,11,12,16 -18,20,21]. | **Guidelines**  • Cochrane Methods  https://methods.cochrane.org/rapidreviews/  • Rapid Reviews Rapid Review Guidebook  https://www.nccmt.ca/uploads/media/media/0001/02/800fe34eaedbad09edf80ad5081b9291acf1c0c2.pdf  **Study example:**  Adherence, barriers and facilitators in the treatment of arterial hypertension: rapid evidence review  https://iris.paho.org/bitstream/handle/10665.2/57367/v47e672023.pdf?sequence=1&isAllowed=y |
| 3. Summary of abstracts | Thematic categorization derived from the abstracts of the included scientific studies [7,20]. | Utilized when decision-makers require rapid evidence to inform immediate actions, with a typical turnaround time of 10 to 15 working days [7,20]. | 1. Definition of the research question  2. Definition of methodological shortcuts as limitations to the scope of the research question  3. Limited literature research  4. Results report  5. Reviewing the report  6. Final report published or sent to the applicant [7,20] | Summarization of endpoints and findings from abstracts [7,20]. | **Study example**  Summary of abstracts about costs of an oral health team in Brazil  https://docs.bvsalud.org/biblioref/2021/01/1146187/sumario-de-resumos-sobre-custos-de-uma-equipe-de-saude-bucal-no-brasil.pdf |
| 4. Evidence maps | It is a structured table with columns and rows on the types of interventions and relevant results in a specific sector or thematic area [1,13,22,24–27]. | As a knowledge translation tool for decision-making and the design of specific interventions, whether effective or ineffective, this approach enables the identification of areas and themes requiring further high-quality research. It facilitates evidence-informed decision-making by presenting findings in an accessible format [1,13,22,24–27]. | 1. Definition of the research question  2. Scope by acronyms  3. Checking existing and ongoing maps to avoid duplication  4. Definition of the structure of row and column headings and filters (intervention categories and result domains).  5. Definition of the name given to the category or subcategory on the map  6. Consultation with interested parties  7. Drawing up a protocol  8. Research and screening  9. Data extraction  10. Critical assessment  11. Coding  12. Data review  13. Map construction [24–27] | This approach employs systematic review methods in a descriptive format, utilizing a structured coding tool to capture detailed information on interventions, outcomes, outcome measurements, study types, populations, geographic scope, and other relevant data. The quality of included systematic reviews is also rigorously assessed [24–27]. | **Guidelines:** Guidance for producing a Campbell evidence and gap map  https://onlinelibrary.wiley.com/doi/10.1002/cl2.1125  **Study example:**  Evidence Map for the Prison System  https://repositorio.enap.gov.br/handle/1/4803  Available platforms  • 3ie https://www.3ieimpact.org/evidence-hub/evidence-gap-maps |
| 5. Technical-scientific report | Synthesis that uses methodological shortcuts for quick answers, with economic information, regulatory aspects and a recommendation for or against the use of a health technology, to support decision-making by managers [7,19]. | Designed to provide decision-makers with rapid yet rigorous access to scientific evidence, with a specific focus on health technology assessment. This approach is employed for the comprehensive analysis of health technologies [7,19]. | 1. Definition of the research question  2. Scope delimitation  3. Team selection  4. Planning  5. Schedule of activities  6. Explanation of methodology  7. Summarizing the findings  8. Interpretation  9. Recommendation  10. Preparation of a technical data sheet for the technology [7,19]. | It is recommended to extract the following information: the type of technology under assessment; the purpose of the technology (prevention, diagnosis, therapy, rehabilitation); basic characteristics; intended uses; various indications; contraindications; and known risks as described in the relevant literature. Additionally, the mechanisms of action of the technology, pertinent safety information, and the current phase of the technology's life cycle (incorporation, initial diffusion, widespread diffusion, abandonment, or exclusion) should be documented [7,19]. | **Guidelines**  Methodological guidelines: drafting technical-scientific opinions  <https://www.gov.br/conitec/pt-br/midias/artigos_publicacoes/diretrizes/diretrizes_metodologicas_ptc.pdf>  **Study example**  Technical-Scientific Opinion - Ticagrelor in Acute Coronary Syndrome  <https://docs.bvsalud.org/biblioref/2018/05/884362/parecer-tecnico-cientifico-ticagrelor-em-sindrome-coronariana-aguda.pdf>  **Available platforms**  Information System of the Brazilian Health Technology Assessment Network (SisRebrats) |
| 6. Critical review | Extensive analysis of scientific articles using appropriate criteria, such as strengths or weaknesses of a specific topic [1,5,13,37,39]. | To make a judgment about a topic by evaluating concepts, usually resulting in the formation of a hypothesis, new concepts or models [1,5,13,37,39]. | 1. There is no formal requirement to present search, synthesis and analysis methods explicitly  2. Critical reading of texts  3. Data extraction and analysis  4. Construction of the report [5]. | Guided by the previously chosen criteria, the synthesis may include a variety of presentation formats, including the conceptual contribution to support existing theory or create new theory [5,39]. | **Study example**  "Mais Médicos" (More Doctors) Program: a critical review of implementation from the perspective of access and universalization of health care  https://www.scielo.br/j/csc/a/yXM6mFq3xDhBPNQCpGRDpBv/abstract/?lang=pt |
| 7. Scoping review | Building an overview of a broad topic by identifying key concepts or concepts [1–4 ,7,13,15,23,26–30,32–40]. | To map and articulate the knowledge and gaps on key concepts, derived from a variety of sources, such as research results, gray literature, and expert opinion. It could serve as a starting point for future research. Scope reviews do not investigate the risk of bias of the included evidence; therefore, there is no guarantee of the quality of the evidence and no clinical recommendations can be made based on the veracity of the review's findings [1–4 ,13,15,23,26–30,32–40]. | 1. Definition of a well-defined theme  2. Definition of the research question  3. Elaboration of a protocol with eligibility criteria and search for evidence  4. Consultation with librarians and researchers who are specialists in the subject matter during the protocol development phase  5. Database and reference list searches  6. Study selection  7. Data extraction  8. Data analysis  9. Presentation of results  10. Evidence summarization [2-4 ,7,13,15,23,26,28–30,32–38,40]. | It can be called a "data graphic", providing the reader with a logical and descriptive summary of the results that aligns with the objectives and questions of the review.  Two steps must take place before the data is formally extracted: (1) in the protocol development phase, develop the extraction form; (2) pilot test the form, or calibration, with two or more reviewers to ensure consistency.  Extraction by a pair of independent reviewers, with discrepancies resolved by a third reviewer.  Some important information that can be mapped: authors, year of publication, origin/country where the source was published or conducted, objectives, population and sample size, methods, types of intervention and details of it, results, and main findings related to the research question.  Suggested secure platform for this stage: Software Research Electronic Data Capture (REDCap) [2-4 ,7,26,29,32,33,37,38] | **Guidelines**  • JBI Manual for Evidence Synthesis  https://jbi-global-wiki.refined.site/space/MANUAL  **Study example**  Evolution of Brazilian human health policies for the prevention and control of antimicrobial resistance: a scope review  https://iris.paho.org/handle/10665.2/57450  Learn More  • Checklist of items: PRISMA for scope reviews  http://www.prisma-statement.org/documents/PRISMA-ScR-Fillable-Checklist_11Sept2019.pdf |
| 8. Review of mixed methods | This approach leverages the findings from both qualitative and quantitative studies, emphasizing the search for empirical evidence, as well as the screening and interpretation of that evidence [1,5,13,14,22,40,46,58–61]. | To evaluate the effectiveness of interventions, along with their feasibility and appropriateness for a specific context, thereby maximizing the potential of evidence to inform policy and practice [1,5,13,14,22,40,46,58–61]. | 1. Elaboration of the research question  2. Protocol development  3. Elaboration of the search strategy with two questions: one for qualitative review and another for quantitative review  4. Selection of studies according to inclusion criteria  5. Assessment of studies and data extraction  6. Synthesis of data for each approach (quantitative and qualitative)  7. Final synthesis of the review by converting all numerical results into statements [1,5,14,40,58,93] | Methodology based on the synthesis of quantitative and qualitative evidence. Evidence can be confirmed, refuted or complemented with one another [1,5,14,40,58,60]. | **Guidelines**  • Mixed Methods Article Reporting Standards  https://apastyle.apa.org/jars/mixed-methods  • JBI Manual for Evidence Synthesis  https://jbi-global-wiki.refined.site/space/MANUAL  **Study example**  Sociodemographic aspects, barriers, and motivations of cyclists in a southern Brazilian city: a mixed methods study  https://www.scielo.br/j/csc/a/5Rh4dTpcFWBLyzX9hbgkNzQ/abstract/?lang=pt |
| 9. Economic assessment review | Data synthesis is conducted to identify the best estimates of health outcomes, taking into account both costs and health impacts. [7,67,86]. | This analysis focuses on the prioritization of interventions, budgetary impact, and resource allocation, while considering the performance of specific technologies [7,67,86]. | 1. Elaboration of the research question  2. Survey of previous studies, development of the justification and objectives  3. Definition of the study population  4. Definition of the study design  5. Definition of the type of economic analysis to be used  6. Description of the interventions to be compared  7. Results  8. Discussion  9. Recommendations  [7,86]. | The same steps may be followed as in systematic reviews [7,86]. | Guidelines  • Economic Evaluation Guideline  https://bvsms.saude.gov.br/bvs/publicacoes/diretrizes_metodologicas_diretriz_avaliacao_economica.pdf  • JBI Manual for Evidence Synthesis  https://jbi-global-wiki.refined.site/space/MANUAL  **Study example**  Pediatric and end-of-life palliative care: a systematic review of economic assessment in health  <https://www.scielo.br/j/rpp/a/yx3xhgXxQP4cRNXZWzBr74r/abstract/?lang=pt> |
| 10. Review of systematic reviews | Synthesis that allows for examining different interventions for the same condition, the same intervention for different conditions, or the same intervention for the same condition but focused on different outcomes. The topic to be investigated should already be widely studied with publications of systematic reviews. The search methods largely resemble those used in a systematic review [1,13–15,32,33,37,40,48–51, 91]. | To document common challenges in conducting studies on the topic of interest and determine whether existing guidelines can help researchers overcome these challenges [1,13–15,32,33,37,40,48–51,91]. | 1. Scope specification  2. Development and registration of a protocol  3. Search for systematic reviews (potentially primary studies)  4. Selection of reviews for inclusion (potentially primary studies)  5. Assessment of the quality of included reviews  6. Collection and presentation of data, descriptive characteristics of the included reviews  7. Collection and presentation of data on the quality of the primary studies included in the systematic reviews  8. Collection, analysis, and presentation of outcome data  9. Assessment of the quality of the body of evidence  10. Interpretation of outcome data and conclusions  11. Overview update [13–15,32,33,37,40,48–51, 91]. | The available guidelines recommend two methods for data analysis and presentation: summarizing the data as they were originally presented in the systematic reviews or reanalyzing the data in a different way than was conducted. A brief summary of the evidence should be made available, accessible and easy to share [13–15,32,33,37,40,48–51,91] | **Guidelines**  • JBI Manual for Evidence Synthesis  https://jbi-global-wiki.refined.site/space/MANUAL  • Cochrane  https://training.cochrane.org/handbook/current/chapter-v#section--2  **Study example**  Efficacy and safety of resistance training after breast cancer surgery: a review of systematic reviews  https://ninho.inca.gov.br/jspui/handle/123456789/7779 |
| 11. Proofreading and opinion | Synthesis of expert opinion narratives, standards for clinical care, consensus guidelines, expert consensus, narrative case report, published discussion documents, conference proceedings or government policy reports. It is considered a legitimate source of evidence, especially when there are no other sources of research on the topics in question [42,67,87,88]. | To complement empirical evidence, since much clinical evidence is produced from the clinical experiences of professionals [42,67,87,88]. | 1. Definition of the objectives/questions of the review  2. Definition of inclusion criteria  3. Definition of the search strategy  4. Assessment of methodological quality  5. Protocol  6. Textual data extraction  7. Data synthesis  8. Results  9. Discussion  10. Conclusions  11. References.  12. Appendices [87] | Double extraction of textual data is recommended. Textual data extraction incorporates various fields related to the type of text, its authors and participants, stated position, context, geographical context, cultural context, logic of the argument, the author's conclusions and the reviewer's comments [87] | **Guidelines**  • JBI Manual for Evidence Synthesis  https://jbi-global-wiki.refined.site/space/MANUAL    **Study example**  School-based mental health promotion: A global policy review  https://pubmed.ncbi.nlm.nih.gov/37139309/ |
| 12. Integrative review | Systematized review that combines results from different sources of quantitative and qualitative information, and then integrates the results into a new review of a mixed nature, providing a deeper understanding of the phenomenon or health issue. It can include empirical and theoretical publications [5,13,22,31,40,46,47,52–57]. | Used to describe and aggregate predefined concepts and results to inform decisions. It enables the synthesis of the state of knowledge on a specific topic, as well as identifies knowledge gaps that need to be addressed through further studies [5,13,22,31,40,46,47,52–57]. | 1. Identification of the problem  2. Literature search, using a comprehensive search strategy;  3. Assessment of data with a focus on authenticity, methodological quality, value of information and representativeness of the primary studies included;  4. Data analysis, through data reduction, display, comparison and conclusions;  5. Presentation of a synthesis of the findings, in a model that portrays the integration process and describes the implications for practice, policy and research, as well as the limitations of the review [5,22,40,47,52–57]. | Generally, the data should cover the study sample (subjects), objectives, methodology used, results and the main findings of each study. [5,22,31,40,47,52–57]. | **Study example**  Realist assessment for health programs and services: an integrative review of the theoretical-methodological literature  <https://www.revistas.usp.br/rlae/article/view/186305> |
| 13. Narrative review | Synthesis of the literature on the topic using informal methods to select and interpret information, without specifying the methodologies used to search, summarize, and organize the information.  Analysis of bibliographic production in a specific subject area, within a specific time frame in order to obtain an overview [1,5,13,22,28,37,39,41–46,54]. | These elements are used in the introduction of a study to justify the problem being addressed. Despite the limited rigor involved, they highlight the need for further investigation into previously unexplored areas of interest. Additionally, there are specific categories for different nomenclatures:  -Historical: Documents the development of research in a specific area.  -Questioner: Presents a topic based on the analysis and synthesis of various studies, requiring intellectual maturity.  -Expository: Explores a topic through the analysis and synthesis of various studies, also necessitating intellectual maturity [1,5,13,22,37,39,41–46,54]. | The methods used to research, summarize and organize the information are not clearly stated [39,43,45]. | There is no specification [39,43,45]. | **Study example**  Covid-19 and the repercussions on mental health: a narrative literature review.  https://www.scielo.br/j/rgenf/a/YD6WWBggJmkcBY8jNsFypSd/?lang=pt |
| 14. Realist review | Attempts to explain how complex interventions and programs work in certain contexts and environments or why they fail [5,39,89,90,94,95]. | To identify and analyze interventions or programs by considering their complexity and the various factors involved, utilizing an explanatory approach guided by theory and a qualitative mixed-methods framework. This analysis seeks to uncover the reasons behind the success or failure of interventions, thereby supporting the development and understanding of complex social interventions [5,39,89, 90,94,95]. | 1. Team formation  2. Background to the topic and location of theoretical bases  3. Title  4. Abstract  5. Introduction  6. Objectives and focus of the review  7. Methods  8. Results  9. Discussion  10. Conclusion and recommendations  11. Funding and conflict of interest  12. Disclosure [5,89, 90, 94]. | It should describe and justify the data or information extracted from the included documents; progressively focus on the main theories of the program, refining the inclusion criteria in the light of the new data. Creating a list of theories, grouping, categorizing or synthesizing theories, drawing up a theoretical framework with the identified evidence [5,89, 90, 94]. | Study example:  Educational practices for the prevention of sexually transmitted infections in adolescence: a realist review  https://pesquisa.bvsalud.org/portal/resource/pt/biblio-1401139  **Learn More**  • Realist synthesis - Rameses training materials  https://www.ramesesproject.org/media/Realist_reviews_training_materials.pdf |
| 15a. Quantitative systematic  review | It is considered the gold standard for researching, gathering, evaluating and summarizing the best available evidence on a clinical question. Characteristics: (a) a clearly defined set of objectives and explicit and reproducible methodology; (b) a well-defined research strategy to identify all studies that would meet the eligibility criteria; (c) extraction of quantifiable information; (d) systematic grouping and synthesis of the results in an orderly manner according to categories; and (e) systematic presentation and synthesis of the characteristics and findings of the included studies [1,15,19,27,40,41,46,47,62,62–67,68 –84,93]. | Used when policymakers need to make decisions to improve health results and the cost-effectiveness of interventions and programs. There are some specific purposes, depending on the topic of the synthesis.  • Prevalence/incidence: To determine the prevalence and/or incidence of a particular condition (local, national or global).  • Accuracy of the diagnostic test: To determine how well a diagnostic test works in terms of sensitivity and specificity for a specific diagnosis.  • Etiology and/or risk: To determine whether and to what extent there is an association between exposure and health outcome, determine the association between specific exposures/risk factors and outcomes [1,15,19,27,40,41,43,46,47,62–84,93]. | 1. Establishing a group of researchers  2. Definition of the clinical question;  3. Creation of protocols and the registering on digital platforms  4. Search (databases, descriptors and search strategies)  5. Screening with selection/exclusion of studies and eligibility  6. Data extraction  7. Assessment of study quality, risk of bias and exploitation of heterogeneity  8. Interpretation of results  9. Tabulation and presentation  10. Conclusions and recommendations [6,15,27,43,47,62–64,66–84,93]. | The extracted data should be presented in a table in the results section of the systematic review. The review may or may not include a meta-analysis (a statistical analysis that combines or groups the results of several independent clinical trials to arrive an estimate of the average effect size attributable to a particular intervention presented in the same metric) [15,19,27,43,47,62,64,65 –84,93]. | **Guidelines**  • JBI Manual for Evidence Synthesis  https://jbi-global-wiki.refined.site/space/MANUAL  • Cochrane  https://training.cochrane.org/handbook/current/chapter-ii  • CRD's guidance for undertaking reviews in health care  https://www.york.ac.uk/media/crd/Systematic_Reviews.pdf  **Study example**  Effectiveness of educational interventions in the prevention of respiratory infections: systematic review and meta-analysis  https://www.scielo.br/j/reben/a/8LPhSyqPzTyTfRmxYVvXkpb/abstract/?lang=pt |
| 15b. Qualitative systematic  review | Integrates and compares the results of qualitative studies. The result can lead to the development of a new theory or an interpretative translation. The objective is to integrate the findings into a broader category, unlike meta-analysis, which aggregates results from studies [1,14,34,46,67,85]. | To investigate the experience or meaning of a particular phenomenon, based on qualitative data [1,14,34,46,85,96]. | 1. Definition of the qualitative research question;  2. Search (databases, descriptors and search strategies)  3. Screening with selection/exclusion of studies and eligibility  4. Data extraction  5. Assessing the quality of studies  6. Interpretation of results  7. Tabulation and presentation  8. Conclusions and recommendations [6,14,34,46,85]. | Meta-aggregation aims to synthesize qualitative evidence comprising data expressed in terms of meanings or experiences of actions or events.  • Meta-synthesis: evaluates, interprets and transforms the findings of qualitative studies to synthesize new conceptualizations and interpretations that could be used for evidence-based medicine.  • Meta-ethnography aggregates written interpretative reports  • Meta-study: a form of meta-synthesis based on three analytical phases: metatheory, metamethod and meta-analysis of data.  • Qualitative Meta-Summary: reflects a form of quantitative logic and can be understood as an aggregation-oriented method, that reflects a view of language as a neutral means of communication [14,34,46,85]. | **Guidelines**  • JBI Manual for Evidence Synthesis  https://jbi-global-wiki.refined.site/space/MANUAL  • Campbell  • https://www2.cifor.org/ebf/wp-content/uploads/sites/32/2017/pubs/Campbell_Collaboration.pdf  **Study example**  The Therapeutic Itinerary in Brazil: a systematic review and meta-synthesis based on negative and positive conceptions of health  https://www.scielo.br/j/sdeb/a/THvRbrVLKYtqLydhYcrthfQ/abstract/?lang=pt  Available platforms  • Evidence synthesis tools  https://training.cochrane.org/handbook/current/chapter-ii  Available Courses  • JBI Comprehensive Systematic Review Training Program (CSRTP)  http://www.ee.usp.br/jbibrasil/cursos/comprehensive-systematic-review-training-program-csrtp/ |
| 15c. Other types of systematic review | It focuses on methodologies, tests and updates and not on quantitative or qualitative studies. These are summaries that incorporate updates from reviews [1,5,13–15,27,39–43,55,64–67,70,71,74–76,78,79,82–84,93,95,97,98]. | - Psychometric: To evaluate the psychometric properties of a specific test, usually to determine the reliability and validity of a specific test or assessment.   • Prognostic: To evaluate the quality/characteristics of health assessment instruments to determine the best tool (in terms of validity, reliability, responsiveness, etc.) for use in practice, for a specific condition or factor. Previously conducted, with the objective of recording the entire process involving the SR, as well as to define the analyses that will be performed, to reduce systematic errors and biases.  • Methodological: To examine methodological issues related to the design, conduct and review of research studies and evidence synthesis. Examine and investigate current research methods and potentially the impact on research quality  • Living syntheses of evidence: Linking evidence and practice in a transparent way for evidence producers, policy makers, guideline developers, funders and publishers [1,5,13–15,27,39–43,55,64–67,70,71,74–76,78,79,82–84,93,95,98,99]. | Stages of traditional systematic reviews  1. Formulating the question  2. Location and selection of studies  3. Critical assessment of studies  4. Data collection  5. Data analysis and presentation  6. Data interpretation  7. Improving and updating the review  [1,5,13–15,27,39–43,55,64–67,70,71,74–76,78,79,82–84,93,95,98,99]. | Should specify data that can answer the specific research question and the objectives of the review [1,5,13–15,27,39–43,55,64–67,70,71,74–76,78,79,82–84,93,95,98,99]. | **Available guidelines**  • Guidance for the production and publication of Cochrane living systematic reviews  https://community.cochrane.org/review-production/production-resources/living-systematic-reviews  **Study example**  Use of anticoagulants in COVID-19 patients: a live systematic review and meta-analysis  https://www.scielo.br/j/jbpneu/a/cM8WVtDJSVcZD4nH86sXDpD/abstract/?format=html&lang=pt&stop=next |
| 16. Evidence synthesis | This approach presents options for addressing a problem to inform decision-makers in the formulation of policies. Based on the results of systematic reviews, it identifies strategies for tackling the issue, conducts equity analyses, and examines implementation considerations. The summary can be derived from the comprehensive evidence synthesis document, while the quick form employs methodological shortcuts to minimize preparation time. [7,19,92]. | Intended for government policymakers, health workers, service users, federal, state, and municipal managers, as well as other stakeholders involved in the decision-making process [7,19,92]. | 1. Prioritization and problem identification  2. Confirmation of synthesis need  3. Definition of the research question  4. Definition of eligibility criteria  5. Filling in the research protocol  6. Research for evidence  7. Selection of eligible studies  8. Data extraction  9. Assessment of the methodological quality of the included studies  10. Elaboration and description of options  11. Equity analysis of options  12. Considerations for implementing the options  13. Drafting the summary  14. Performing a policy dialogue  15. Updating the post-dialog synthesis  16. Merit review  17. Synthesis update after merit review [7,19,92]. | Includes describing the data in order to identify options to address the central problem of the synthesis [7,19,92]. | **Available guidelines**  Methodological guideline: synthesis of evidences for policy  https://bvsms.saude.gov.br/bvs/publicacoes/diretriz_sintese_evidencias_politicas.pdf  **Study example**  Evidence synthesis for health policies: promoting early childhood development  https://bvsms.saude.gov.br/bvs/publicacoes/sintese_evidencias_politicas_primeira_infancia.pdf |

References

1. Grant MJ, Booth A. A typology of reviews: an analysis of 14 review types and associated methodologies. Health Info Libraries J. 2009;26: 91–108. doi:10.1111/j.1471-1842.2009.00848.x

2. Sarrami-Foroushani P, Travaglia J, Debono D, Clay-Williams R, Braithwaite J. Scoping Meta-Review: Introducing a New Methodology. Clinical and Translational Science. 2015;8: 77–81.

3. Arksey H, O’Malley. International Journal of Social Research Methodology: Theory & Practice. Scoping Studies: Towards a Methodological Framework. 8th ed. 2005: 19–32.

4. Peters MDJ, Godfrey C, McInerney P, Khalil H, Larsen P, Marnie C, et al. Best practice guidance and reporting items for the development of scoping review protocols. JBI evidence synthesis. 2022.

5. Tricco AC, Soobiah C, Antony J, Cogo E, Macdonald H, Lillie E, et al. A scoping review identifies multiple emerging knowledge synthesis methods, but few studies operationalize the method. Journal of Clinical Epidemiology. 2016;73: 19–28.

6. Page MJ, McKenzie JE, Bossuyt PM, Boutron I, Hoffmann TC, Mulrow CD, et al. The PRISMA 2020 statement: an updated guideline for reporting systematic reviews. BMJ. 2021; n71. doi:10.1136/bmj.n71

7. Brasil. Serviço de produção de evidências para apoio à tomada de decisão : portfólio de produtos. Ministério da Saúde. Secretaria de Ciência, Tecnologia, Inovação e Insumos Estratégicos em Saúde. Departamento de Ciência e Tecnologia; 2019. Available: https://bvsms.saude.gov.br/bvs/publicacoes/servico_producao_apoio_evidencias_tomada_decisao_portifolio_produtos.pdf

8. Hartling L, Guise J-M, Kato E, Anderson J, Aronson N, Belinson S, et al. EPC Methods: An Exploration of Methods and Context for the Production of Rapid Reviews. Rockville (MD): Agency for Healthcare Research and Quality (US); 2015. Available: http://www.ncbi.nlm.nih.gov/books/NBK274092/

9. Tapia-Benavente L, Vergara-Merino L, Garegnani LI, Ortiz-Muñoz L, Loézar Hernández C, Vargas-Peirano M. Rapid reviews: definitions and uses. Medwave. 2021;21: e8090–e8090.

10. Haby MM, Chapman E, Clark R, Barreto J, Reveiz L, Lavis JN. What are the best methodologies for rapid reviews of the research evidence for evidence-informed decision making in health policy and practice: a rapid review. Health research policy and systems. 2016;14: 83–83.

11. Tricco AC, Antony J, Zarin W, Strifler L, Ghassemi M, Ivory J, et al. A scoping review of rapid review methods. BMC Med. 2015;13: 224. doi:10.1186/s12916-015-0465-6

12. Silva MT, Silva END, Barreto JOM. Rapid response in health technology assessment: a Delphi study for a Brazilian guideline. BMC Med Res Methodol. 2018;18: 51. doi:10.1186/s12874-018-0512-z

13. Mota De Sousa LM, Furtado Firmino C, Alves Marques-Vieira CM, Silva Pedro Severino S, Castelão Figueira Carlos Pestana H. Revisões da literatura científica: tipos, métodos e aplicações em enfermagem. RPER. 2018;1: 45–55. doi:10.33194/rper.2018.v1.n1.07.4391

14. Sutton A, Clowes M, Preston L, Booth A. Meeting the review family: exploring review types and associated information retrieval requirements. Health information and libraries journal. 2019;36: 202–222.

15. Systematic Reviews: CRD’s guidance for undertaking reviews in health care. Centre for Reviews and Dissemination (CRD); 2009 Jan p. 294. Available: https://www.york.ac.uk/media/crd/Systematic_Reviews.pdf

16. Speckemeier C, Niemann A, Wasem J, Bucherger B, Neusser S. Methodological guidance for rapid reviews in healthcare: a scoping review. Research synthesis methods. 2022.

17. Garritty C, Gartlehner G, Nussbaumer-Streit B, King VJ, Hamel C, Kamel C, et al. Cochrane Rapid Reviews Methods Group offers evidence-informed guidance to conduct rapid reviews. Journal of Clinical Epidemiology. 2021;130: 13–22. doi:10.1016/j.jclinepi.2020.10.007

18. Varker T, Forbes D, Dell L, Weston A, Merlin T, Hodson S, et al. Rapid evidence assessment: Increasing the transparency of an emerging methodology. Journal of Evaluation in Clinical Practice. 2015;21: 1199–1204.

19. Brasil. Diretrizes metodológicas : elaboração de pareceres técnico-científicos. Ministério da Saúde. Secretaria de Ciência, Tecnologia, Inovação e Insumos Estratégicos em Saúde. Departamento de Gestão e Incorporação de Tecnologias e Inovação em Saúde.; 2021 p. 122. Available: https://www.gov.br/conitec/pt-br/midias/artigos_publicacoes/diretrizes/diretrizes_metodologicas_ptc.pdf

20. Smela B, Toumi M, Świerk K, Francois C, Biernikiewicz M, Clay E, et al. Rapid literature review: definition and methodology. Journal of Market Access & Health Policy. 2023;11: 2241234. doi:10.1080/20016689.2023.2241234

21. Garritty C, Hamel C, Trivella M, Gartlehner G, Nussbaumer-Streit B, Devane D, et al. Updated recommendations for the Cochrane rapid review methods guidance for rapid reviews of effectiveness. BMJ. 2024; e076335. doi:10.1136/bmj-2023-076335

22. Gough D, Thomas J, Oliver S. Clarifying differences between review designs and methods. Systematic reviews. 2012;1: 28–28.

23. Thomas A, Lubarsky S, Durning SJ, Young ME. Knowledge Syntheses in Medical Education: Demystifying Scoping Reviews. Academic medicine : journal of the Association of American Medical Colleges. 2017;92: 161–166.

24. Snilstveit B, Vojtkova M, Bhavsar A, Stevenson J, Gaarder M. Evidence & Gap Maps: A tool for promoting evidence informed policy and strategic research agendas. Journal of Clinical Epidemiology. 2016;79: 120–129. doi:10.1016/j.jclinepi.2016.05.015

25. White H, Albers B, Gaarder M, Kornør H, Littell J, Marshall Z, et al. Guidance for producing a Campbell evidence and gap map. Campbell Systematic Reviews. 2020;16: e1125. doi:10.1002/cl2.1125

26. Khalil H, Tricco AC. Differentiating between mapping reviews and scoping reviews in the evidence synthesis ecosystem. Journal of Clinical Epidemiology. 2022;149: 175–182. doi:10.1016/j.jclinepi.2022.05.012

27. Campbell F, Tricco AC, Munn Z, Pollock D, Saran A, Sutton A, et al. Mapping reviews, scoping reviews, and evidence and gap maps (EGMs): the same but different— the “Big Picture” review family. Syst Rev. 2023;12: 45. doi:10.1186/s13643-023-02178-5

28. Khalil H, Peters MD, Tricco AC, Pollock D, Alexander L, McInerney P, et al. Conducting high quality scoping reviews-challenges and solutions. Journal of clinical epidemiology. 2021;130: 156–160.

29. Westphaln KK, Regoeczi W, Masotya M, Vazquez-Westphaln B, Lounsbury K, McDavid L, et al. From Arksey and O’Malley and Beyond: Customizations to enhance a team-based, mixed approach to scoping review methodology. MethodsX. 2021;8. Available: ["https://www.embase.com/search/results?subaction=viewrecord&id=L2012741591&from=export", "http://dx.doi.org/10.1016/j.mex.2021.101375"]

30. Peters MDJ, Godfrey CM, Khalil H, McInerney P, Parker D, Soares CB. Guidance for conducting systematic scoping reviews. International journal of evidence-based healthcare. 2015;13: 141–146.

31. Whittemore R, Knafl K. The integrative review: updated methodology. Journal of advanced nursing. 2005;52: 546–553.

32. Pollock D, Davies EL, Peters MDJ, Tricco AC, Alexander L, McInerney P, et al. Undertaking a scoping review: A practical guide for nursing and midwifery students, clinicians, researchers, and academics. Journal of advanced nursing. 2021;77: 2102–2113.

33. Chambergo-Michilot D, Diaz-Barrera ME, Benites-Zapata VA. Scoping reviews, umbrella reviews and focused mapping review synthesis: Methodological aspects and applications. Revista Peruana de Medicina Experimental y Salud Publica. 2021;38: 136–142.

34. Lockwood C, Munn Z, Porritt K. Qualitative research synthesis: methodological guidance for systematic reviewers utilizing meta-aggregation. International journal of evidence-based healthcare. 2015;13: 179–187.

35. Colquhoun HL, Levac D, O’Brien KK, Straus S, Tricco AC, Perrier L, et al. Scoping reviews: Time for clarity in definition, methods, and reporting. Journal of Clinical Epidemiology. 2014;67: 1291–1294.

36. Pham MT, Rajić A, Greig JD, Sargeant JM, Papadopoulos A, McEwen SA. A scoping review of scoping reviews: advancing the approach and enhancing the consistency. Research synthesis methods. 2014;5: 371–385.

37. Samnani SS, Vaska M, Ahmed S, Turin TC. Review Typology: The Basic Types of Reviews for Synthesizing Evidence for the Purpose of Knowledge Translation. Journal of the College of Physicians and Surgeons--Pakistan : JCPSP. 2017;27: 635–641.

38. Cacchione PZ. The Evolving Methodology of Scoping Reviews. Clinical nursing research. 2016;25: 115–119.

39. Xiao Y, Watson M. Guidance on Conducting a Systematic Literature Review. Journal of Planning Education and Research. 2019;39: 93–112. doi:10.1177/0739456X17723971

40. Whittemore R, Chao A, Jang M, Minges KE, Park C. Methods for knowledge synthesis: An overview. Heart & Lung. 2014;43: 453–461. doi:10.1016/j.hrtlng.2014.05.014

41. Cordeiro AM, Oliveira GMD, Rentería JM, Guimarães CA. Revisão sistemática: uma revisão narrativa. Rev Col Bras Cir. 2007;34: 428–431. doi:10.1590/S0100-69912007000600012

42. Moreira W. Revisão de Literatura e Desenvolvimento Científico: conceitos e estratégias para confecção. Janus. 2004;1: 21–30.

43. Gomes Nazareth CC. Revisão de literatura e revisão sistemática: uma análise objetiva. ijosd. 2020 [cited 21 Nov 2024]. doi:10.22409/ijosd.v0i55.43132

44. Wong G, Greenhalgh T, Westhorp G, Buckingham J, Pawson R. RAMESES publication standards: meta-narrative reviews. BMC Med. 2013;11: 20. doi:10.1186/1741-7015-11-20

45. Green BN, Johnson CD, Adams A. Writing narrative literature reviews for peer-reviewed journals: Secrets of the trade. Journal of Sports Chiropractic and Rehabilitation. 2001;15: 5–17.

46. Sobrido Prieto M, Rumbo-Prieto JM. The systematic review: Plurality of approaches and methodologies. Enfermeria clinica (English Edition). 2018;28: 387–393.

47. Soares L, Campos Verdes Rodrigues ID, Martins LN, Ribeiro Da Silveira FD, Fortes Figueiredo ML. Literature review: particularities of each type of study / Revisão de literatura: particularidades de cada tipo de estudo. Rev Enferm UFPI. 2014;2: 14. doi:10.26694/reufpi.v2i5.1200

48. Gates M, Gates A, Guitard S, Pollock M, Hartling L. Guidance for overviews of reviews continues to accumulate, but important challenges remain: a scoping review. Systematic Reviews. 2020;9. Available: ["https://www.embase.com/search/results?subaction=viewrecord&id=L2007160475&from=export", "http://dx.doi.org/10.1186/s13643-020-01509-0"]

49. Lunny C, Brennan SE, McDonald S, McKenzie JE. Toward a comprehensive evidence map of overview of systematic review methods: Paper 1-purpose, eligibility, search and data extraction. Systematic Reviews. 2017;6. Available: ["https://www.embase.com/search/results?subaction=viewrecord&id=L619329461&from=export", "http://dx.doi.org/10.1186/s13643-017-0617-1"]

50. Hasanpoor E, Hallajzadeh J, Siraneh Y, Hasanzadeh E, Haghgoshayie E. Using the Methodology of Systematic Review of Reviews for Evidence-Based Medicine. Ethiopian journal of health sciences. 2019;29: 775–778.

51. Gentles SJ, Charles C, Nicholas DB, Ploeg J, McKibbon KA. Reviewing the research methods literature: principles and strategies illustrated by a systematic overview of sampling in qualitative research. Systematic reviews. 2016;5: 172–172.

52. Botelho LLR, Cunha CCDA, Macedo M. O método da revisão integrativa nos estudos organizacionais. GeS. 2011;5: 121. doi:10.21171/ges.v5i11.1220

53. Mendes KDS, Silveira RCDCP, Galvão CM. Revisão integrativa: método de pesquisa para a incorporação de evidências na saúde e na enfermagem. Texto contexto - enferm. 2008;17: 758–764. doi:10.1590/S0104-07072008000400018

54. Soares CB, Hoga LAK, Peduzzi M, Sangaleti C, Yonekura T, Silva DRAD. Integrative Review: Concepts And Methods Used In Nursing. Rev esc enferm USP. 2014;48: 335–345. doi:10.1590/S0080-6234201400002000020

55. Russell CL. An overview of the integrative research review. Progress in Transplantation. 2005;15: 8–13.

56. Hopia H, Latvala E, Liimatainen L. Reviewing the methodology of an integrative review. Scandinavian journal of caring sciences. 2016;30: 662–669.

57. Souza MT de, Silva MD da, Carvalho R de. Integrative review: what is it? How to do it? TT - Revisão integrativa: o que é e como fazer. Einstein (Säo Paulo). 2010;8. Available: ["http://apps.einstein.br/revista/arquivos/PDF/1134-Einsteinv8n1p102-106.pdf", "http://apps.einstein.br/revista/arquivos/PDF/1134-Einsteinv8n1_p102-106_port.pdf"]

58. Cardoso V, Trevisan I, Cicolella D de A, Waterkemper R. Systematic review of mixed methods: method of research for the incorporation of evidence in nursing TT - Revisión sistemática de métodos mistos: método de investigación para la incorporación de evidencias en la enfermería TT - Revisão sistemática de métodos mistos: método de pesquisa para a incorporação de evidências na enfermagem. Texto & contexto enferm. 2019;28: e20170279–e20170279.

59. Lizarondo L, Stern C, Judith C, Godfrey C, Rieger K, Salmond S, et al. JBI Manual for Evidence Synthesis. Chapter 8: Mixed methods systematic reviews. JBI; 2020. doi:10.46658/JBIMES-20-01

60. Galvão MCB, Ricarte ILM. Revisão sistemática da literatura: conceituação, produção e publicação. Logeion. 2019;6: 57–73. doi:10.21728/logeion.2019v6n1.p57-73

61. Pearson A, White H, Bath-Hextall F, Salmond S, Apostolo J, Kirkpatrick P. A mixed-methods approach to systematic reviews. International Journal of Evidence-Based Healthcare. 2015;13: 121–131. doi:10.1097/XEB.0000000000000052

62. Ravindran V, Shankar S. Systematic reviews and meta-analysis demystified. 2015;10: 89–94.

63. Bello AK, Wiebe N, Garg AX, Tonelli M. Basics of systematic reviews and meta-analyses for the nephrologist. Nephron - Clinical Practice. 2011;119: c50–c60.

64. Gomes IS, Caminha I de O. Guia para estudos de revisão sistemática: uma opção metodológica para as Ciências do Movimento Humano TT - Guía de revisión sistemática de estudios: una opción para la metodología de las Ciencias del Movimiento Humano TT - Guide to systematic review of studies: an option for the methodology of Human Movement Sciences. Movimento (Porto Alegre). 2014;20: 395–411.

65. Farhat N, Tsaioun K, Saunders-Hastings P, Morgan RL, Ramoju S, Hartung T, et al. Systematic review in evidence-based risk assessment. ALTEX. 2021.

66. Knoll T, Omar MI, Maclennan S, Hernández V, Canfield S, Yuan Y, et al. Key Steps in Conducting Systematic Reviews for Underpinning Clinical Practice Guidelines: Methodology of the European Association of Urology. European Urology. 2018;73: 290–300.

67. Munn Z, Stern C, Aromataris E, Lockwood C, Jordan Z. What kind of systematic review should I conduct? A proposed typology and guidance for systematic reviewers in the medical and health sciences. BMC medical research methodology. 2018;18: 5–5.

68. Noordzij M, Hooft L, Dekker FW, Zoccali C, Jager KJ. Systematic reviews and meta-analyses: when they are useful and when to be careful. Kidney international. 2009;76: 1130–1136.

69. Villasís-Keever MÁ, Rendón-Macías ME, García H, Miranda-Novales MG, Escamilla-Núñez A. Systematic review and meta-analysis as a support tools for research and clinical practice. Revista Alergia Mexico. 2020;67: 62–72.

70. Brizola J, Fantin N. Revisão da literatura e revisão sistemática da literatura. 2017;3. doi:https://doi.org/10.30681/relva.v3i2.1738

71. Sampaio R, Mancini M. Estudos de revisão sistemática: um guia para síntese criteriosa da evidência científica. Rev bras fisioter. 2007;11: 83–89. doi:10.1590/S1413-35552007000100013

72. Cook DJ, Sackett DL, Spitzer WO. Methodologic guidelines for systematic reviews of randomized control trials in health care from the potsdam consultation on meta-analysis. Journal of Clinical Epidemiology. 1995;48: 167–171. doi:10.1016/0895-4356(94)00172-M

73. Higgins J, Thomas J, Chandler J, Cumpston M, Li T, Page M, et al. Cochrane Handbook for Systematic Reviews of Interventions. 2024. Available: www.training.cochrane.org/handbook.

74. Tawfik GM, Dila KAS, Mohamed MYF, Tam DNH, Kien ND, Ahmed AM, et al. A step by step guide for conducting a systematic review and meta-analysis with simulation data. Trop Med Health. 2019;47: 46. doi:10.1186/s41182-019-0165-6

75. Wright RW, Brand RA, Dunn W, Spindler KP. How to Write a Systematic Review: Clinical Orthopaedics and Related Research. 2007;455: 23–29. doi:10.1097/BLO.0b013e31802c9098

76. Jones P, Ewan H, Lane T, Adshead J, Vasdev N, Rai BP. How to write a systematic review: Tips and tricks for surgeons in training. Journal of Clinical Urology. 2017;10: 148–153.

77. Molina Arias M. Aspectos metodológicos del metaanálisis (2) TT - Methodological aspects of meta-analysis (2). Pediatr aten prim. 2018;20: 401–405.

78. Krnic Martinic M, Pieper D, Glatt A, Puljak L. Definition of a systematic review used in overviews of systematic reviews, meta-epidemiological studies and textbooks. BMC medical research methodology. 2019;19: 203–203.

79. Mohamed Shaffril HA, Samsuddin SF, Abu Samah A. The ABC of systematic literature review: the basic methodological guidance for beginners. Qual Quant. 2021;55: 1319–1346. doi:10.1007/s11135-020-01059-6

80. Johnson BT, Hennessy EA. Systematic reviews and meta-analyses in the health sciences: Best practice methods for research syntheses. Social Science & Medicine. 2019;233: 237–251. doi:10.1016/j.socscimed.2019.05.035

81. Tufanaru C, Munn Z, Aromataris E, Campbell J, Hopp L. Chapter 3: Systematic reviews of effectiveness. JBI Manual for Evidence Synthesis. JBI; 2020. doi:10.46658/JBIMES-20-04

82. Rutter D, Francis J, Coren E, Fisher M. SCIE systematic research reviews: guidelines. Social Care Institute for Excellence; 2006 p. 122. Available: https://lx.iriss.org.uk/sites/default/files/resources/rr01.pdf

83. Brasil. Diretrizes metodológicas: elaboração de revisão sistemática e metanálise de estudos observacionais comparativos sobre fatores de risco e prognósticos. Ministério da Saúde. Secretaria de Ciência, Tecnologia e Insumos Estratégicos. Departamento de Ciência e Tecnologia; 2014 Apr.

84. NICE. The guidelines manual. National Institute for Health and Care Excellence - NICE; 2012 Nov p. 218. Available: https://www.nice.org.uk/process/pmg6/resources/the-guidelines-manual-pdf-2007970804933

85. Wanden-Berghe C, Sanz-Valero J. Systematic reviews in nutrition: Standardized methodology. British Journal of Nutrition. 2012;107: S3–S7.

86. Gomersall J, Jadotte Y, Xue Y, Lockwood S, Riddle DP. Chapter 8: Systematic reviews of economic evidence. JBI Manual for Evidence Synthesis. JBI; 2020. doi:10.46658/JBIMES-20-01

87. McArthur A, Klugárová J, Yan H, Florescu S. Innovations in the systematic review of text and opinion. International journal of evidence-based healthcare. 2015;13: 188–195.

88. McArthur A, Klugarova J, Yan H, Florescu S. Chapter 4: Systematic reviews of text and opinion. JBI Manual for Evidence Synthesis. JBI; 2020. doi:10.46658/JBIMES-20-05

89. Pawson R, Greenhalgh T, Harvey G, Walshe K. Realist review - a new method of systematic review designed for complex policy interventions. J Health Serv Res Policy. 2005;10: 21–34. doi:10.1258/1355819054308530

90. Yonekura T, Quintans JR, Soares CB, Negri Filho AAD. Realist review as a methodology for using evidence in health policies: an integrative review. Revista da Escola de Enfermagem da U S P. 2019;53: e03515–e03515.

91. Iannizzi C, Akl EA, Kahale LA, Dorando E, Mosunmola Aminat A, Barker JM, et al. Methods and guidance on conducting, reporting, publishing and appraising living systematic reviews: A scoping review protocol. F1000Research. 2021;10. Available: ["https://www.embase.com/search/results?subaction=viewrecord&id=L637201001&from=export", "http://dx.doi.org/10.12688/f1000research.55108.1"]

92. Felt E, Carrasco JM, Vives-Cases C. Methodology for the development of policy brief in public health]. Gaceta sanitaria. 2018;32: 390–392.

93. Galvao MCB, Carmona F, Grand R, Pluye P, Ricarte ILM, Pilotto BS, et al. Disseminating health evidence summaries to increase evidence use in health care. Revista de Saúde Pública. 2018;52: 547–563. doi:10.1590/0101-6628.123

94. Yonekura T, Quintans JR, Soares CB, Negri Filho AAD. Realist review as a methodology for using evidence in health policies: an integrative review. Revista da Escola de Enfermagem da U S P. 2019;53: e03515–e03515.

95. Samnani SS, Vaska M, Ahmed S, Turin TC. Review Typology: The Basic Types of Reviews for Synthesizing Evidence for the Purpose of Knowledge Translation. Journal of the College of Physicians and Surgeons--Pakistan : JCPSP. 2017;27: 635–641.

96. Munn Z, Aromataris E, Tufanaru C, Stern C, Porritt K, Farrow J, et al. The development of software to support multiple systematic review types: the Joanna Briggs Institute System for the Unified Management, Assessment and Review of Information (JBI SUMARI). International journal of evidence-based healthcare. 2019;17: 36–43.

97. Needleman IG. A guide to systematic reviews. Journal of clinical periodontology. 2002;29: 6–8.

98. Deeks J, Bossuyt P, Leeflang M. Cochrane Handbook for Systematic Reviews of Diagnostic Test Accuracy. [cited 19 Nov 2024]. Available: https://training.cochrane.org/handbook-diagnostic-test-accuracy

99. Needleman IG. A guide to systematic reviews. Journal of clinical periodontology. 2002;29: 6–8.
