# Supplementary material for: Scoping review of evidence synthesis: Concepts, types and methods
Source: PLoS One. 2025 May 16;20(5):e0323555. doi: 10.1371/journal.pone.0323555 (PMC12084050; doi:10.1371/journal.pone.0323555)
Supplement: S3 Appendix — (DOCX) [file pone.0323555.s003.docx]

**S3 Appendix Excluded studies and their reasons.**

| **Authors** | **Study title** | **Year of publication** | **Journal** | **Reason for exclusion** | |
| --- | --- | --- | --- | --- | --- |
| Turner JR, Durham TA[100] | Meta-Methodology: Conducting and Reporting Meta-Analyses | 2014 | Journal of Clinical Hypertension | Did not report concepts, method and type of synthesis | |
| Varandas T, Carneiro AV[101] | Types of clinical studies. Systematic reviews. | 2006 | Revista portuguesa de cardiologia | Did not report concepts, method and type of synthesis | |
| Pearson A, White H, Bath-Hextall F, Salmond S, Apostolo J, Kirkpatrick  P [102] | A mixed-methods approach to systematic reviews | 2015 | International Journal of Evidence-Based Healthcare | Did not report concepts, method and type of synthesis | |
| Petropoulou M, Efthimiou O, Rücker G, Schwarzer G, Furukawa TA, Pompoli A, *et al.* [103] | A review of methods for addressing components of interventions in meta-analysis | 2021 | PLoS ONE | Did not report concepts, method and type of synthesis | |
| Arias MM [104] | Aspectos metodológicos del metaanálisis | 2018 | Pediatría Atención Primaria | Did not report concepts, method and type of synthesis | |
| Khan S, Memon MA [105] | Meta-analysis: a critical appraisal of the methodology, benefits and drawbacks. | 2019 | British journal of hospital medicine | Did not report concepts, method and type of synthesis | |
| Dixon-Woods M, Agarwal S, Jones D, Young B, Sutton A [106] | Synthesising qualitative and quantitative evidence: a review of possible methods | 2005 | Journal of Health Services Research & Policy | Did not report concepts, method and type of synthesis | |
| Barnett-Page E, Thomas J [107] | Methods for the synthesis of qualitative research: a critical review | 2009 | BMC Medical Research Methodology | Did not report concepts, method and type of synthesis | |
| Sandelowski M, Voils CI, Leeman J, Crandel JL [108] | Mapping the Mixed Methods–Mixed Research Synthesis Terrain | 2012 | Journal of mixed methods research | Did not report concepts, method and type of synthesis | |
| Gough D [109] | Meta-narrative and realist reviews: Guidance, rules, publication standards and quality appraisal | 2013 | BMC Medicine | Did not report concepts, method and type of synthesis |  |
| Hannes K, Lockwood C [110] | Pragmatism as the philosophical foundation for the Joanna Briggs meta-aggregative  approach to qualitative evidence synthesis. | 2011 | Journal of advanced nursing | Did not report concepts, method and type of synthesis |  |
| Garritty C, Hamel C, Trivella M, Gartlehner G, Nussbaumer-Streit B, Devane D, *et al.* [111] | Cochrane Rapid Reviews Interim Guidance from the Cochrane Rapid Reviews Methods . | 2020 | Cochrane Rapid Reviews Methods Group | Outdated version |  |
| Smela B, Toumi M, Świerk K, Francois C, Biernikiewicz M, Clay E, *et al.* [112] | Definition and Methodology of Rapid Literature Reviews | 2023 | Value Health | Congress abstract |  |
| Su R, Yu X, Shi Q, Liu X, Sun Y, Lan H, et al.[113] | Current Situation and Progress of Evidence Synthesis Methodology | 2023 | Medical Journal of Peking Union Medical College Hospital | Chinese language |  |
| Martsevich SY, Navasardyan AR, Lobastov KV, Mikaelyan MV, Mikhaylenko EV, Suvorov A, *et al.* [114] | Systematic review and meta-analysis: a critical examination of the methodology | 2023 | Rational Pharmacotherapy in  Cardiology | Russian language |  |

References

100. Turner JR, Durham TA. Meta-Methodology: Conducting and Reporting Meta-Analyses. Journal of Clinical Hypertension. 2014;16: 91–93.

101. Varandas T, Carneiro AV. Types of clinical studies. Systematic reviews. Revista portuguesa de cardiologia : orgao oficial da Sociedade Portuguesa de Cardiologia = Portuguese journal of cardiology : an official journal of the Portuguese Society of Cardiology. 2006;25: 233–246.

102. Pearson A, White H, Bath-Hextall F, Salmond S, Apostolo J, Kirkpatrick P. A mixed-methods approach to systematic reviews. International Journal of Evidence-Based Healthcare. 2015;13: 121–131. doi:10.1097/XEB.0000000000000052

103. Petropoulou M, Efthimiou O, Rücker G, Schwarzer G, Furukawa TA, Pompoli A, et al. A review of methods for addressing components of interventions in meta-analysis. PLoS ONE. 2021;16. Available: ["https://www.embase.com/search/results?subaction=viewrecord&id=L2011046575&from=export", "http://dx.doi.org/10.1371/journal.pone.0246631"]

104. Molina Ariasa M. Aspectos metodológicos del metaanálisis (1) TT - Methodological aspects of meta-analysis (1). Pediatr aten prim. 2018;20: 297–302.

105. Khan S, Memon B, Memon MA. Meta-analysis: a critical appraisal of the methodology, benefits and drawbacks. British journal of hospital medicine (London, England : 2005). 2019;80: 636–641.

106. Dixon-Woods M, Agarwal S, Jones D, Young B, Sutton A. Synthesising qualitative and quantitative evidence: A review of possible methods. J Health Serv Res Policy. 2005;10: 45–53. doi:10.1177/135581960501000110

107. Barnett-Page E, Thomas J. Methods for the synthesis of qualitative research: a critical review. BMC Med Res Methodol. 2009;9: 59. doi:10.1186/1471-2288-9-59

108. Sandelowski M, Voils CI, Leeman J, Crandell JL. Mapping the Mixed Methods–Mixed Research Synthesis Terrain. Journal of Mixed Methods Research. 2012;6: 317–331. doi:10.1177/1558689811427913

109. Gough D. Meta-narrative and realist reviews: Guidance, rules, publication standards and quality appraisal. BMC Medicine. 2013;11. Available: ["https://www.embase.com/search/results?subaction=viewrecord&id=L52419135&from=export", "http://www.biomedcentral.com/1741-7015/11/22", "http://dx.doi.org/10.1186/1741-7015-11-22"]

110. Hannes K, Lockwood C. Pragmatism as the philosophical foundation for the Joanna Briggs meta-aggregative approach to qualitative evidence synthesis. Journal of advanced nursing. 2011;67: 1632–1642.

111. Garritty C, Gartlehner G, Kamel C, King V, Nussbaumer-Streit B, Stevens A, et al. Cochrane Rapid Reviews. Interim Guidance from the Cochrane Rapid Reviews Methods. Cochrane; 2020 Mar p. 3.

112. Smela B, Toumi M, Swierk K, Francois C, Biernikiewicz M, Clay E, et al. SA51 Definition and Methodology of Rapid Literature Reviews. Value in Health; 2023. p. S551. Available: https://www.valueinhealthjournal.com/article/S1098-3015(23)06091-6/abstract

113. SU R, YU X, Qianling S, LUO X, SUN Y, LAN H, et al. Situation and Progress of Evidence Synthesis Methodology. Medical Journal of Peking Union Medical College Hospital. 2023;14: 1301–1309.

114. Martsevich S.Yu. SYu, Navasardyan AR, Lobastov KV, Mikaelyan MV, Mikhaylenko EV, Suvorov AYu, et al. Systematic review and meta-analysis: a critical examination of the methodology. Racionalʹnaâ farmakoterapiâ v kardiologii. 2023;19: 382–397. doi:10.20996/1819-6446-2023-2923
