# Supplementary material for: Scoping review of evidence synthesis: Concepts, types and methods
Source: PLoS One. 2025 May 16;20(5):e0323555. doi: 10.1371/journal.pone.0323555 (PMC12084050; doi:10.1371/journal.pone.0323555)
Supplement: Attachment — Submitted filename: Response to reviewers.docx [file pone.0323555.s005.docx]

Dear Editorial Board,

We sincerely appreciate the time and effort that the editorial team and reviewers have dedicated to evaluating our manuscript. We have carefully addressed each of the comments and suggestions provided, making the necessary revisions to ensure our manuscript meets PLOS ONE’s standards. Below, we provide detailed responses to each concern raised.

**1. Manuscript Formatting:**
We have revised our manuscript to comply with PLOS ONE's style requirements, following the provided formatting guidelines for the main text and title, authors, and affiliations.

**2. Text Overlap with Previous Publications:**
We have thoroughly reviewed the manuscript for any overlapping text with the publication referenced (https://bmcmedresmethodol.biomedcentral.com/articles/10.1186/s12874-017-0468-4). Any duplicated text outside the methods section has been properly cited, rephrased, or removed as necessary to avoid redundancy.

**3. Grant Information Consistency:**
We have corrected and aligned the grant information across the ‘Funding Information’ and ‘Financial Disclosure’ sections, ensuring accurate and consistent details, including grant numbers.

**4. Copyrighted Figures (Figure 2):**
We have either removed Figure 2 with an alternative that is compliant with the CC BY 4.0 license.

**5. Data Anonymization:**
All personal information has been removed from the dataset to comply with PLOS ONE’s data-sharing policy. We have re-uploaded a fully anonymized dataset, ensuring that no hidden columns contain sensitive information.

**6. Supporting Information Captions:**
Captions for all Supporting Information files have been added at the end of the manuscript, with corresponding in-text citations updated accordingly.

**7. Reference List Review:**
We have reviewed our reference list to ensure completeness and accuracy.

**Responses to Reviewer Comments:**

1. **Definitions and Examples for Review Types:**
   - We have refined and expanded the definitions of different review types, providing clearer distinctions and specific examples to enhance clarity. To address this, we have refined and expanded our definitions, ensuring that each review type is more precisely delineated. Additionally, we have provided concrete examples to illustrate these distinctions, which are included in the appendix table for reference.
   - These revisions aim to enhance clarity and assist readers in understanding the methodological approaches associated with each review type.
2. **Methodological Strengths and Weaknesses of Review Types:**
   - We appreciate the reviewer’s suggestion to provide a more in-depth analysis of the methodological strengths and weaknesses of each review type. In response, we have incorporated a discussion section (lines 159-166, 168-174, 177-184, 187-191, 196, 197, 201-2012, 217-224, 231-233, 237, 243-248, 292-316) detailed critique that outlines the specific advantages, limitations, and best use cases for each type of review. This additional discussion enhances the manuscript’s methodological rigor and provides clearer guidance for researchers on selecting the most appropriate review methodology for their objectives.
   - To maintain clarity and accessibility, we have compiled these methodological considerations into an appendix table, where each review type is systematically analyzed. We believe this format allows readers to quickly compare and contrast different methodologies while ensuring the main text remainsponde focused and concise.
3. **Standardization of Terminology:**
   - The conclusion now includes concrete suggestions for standardizing terminology and methodologies, including a proposed framework and collaboration strategies with key stakeholders (lines 334-344).
4. **Terminology Consistency:**
   - We have ensured that terms such as "rapid review," "systematic map," "scoping review," "narrative review," "realist review," and "integrative review" are clearly defined and consistently used throughout in the results section.
5. **Study Selection Process and Inter-Rater Agreement:**
   - We appreciate the reviewer´s suggestion regarding the calibration of search queries and the assessment of inter-rater agreement during the study selection process. Regarding the search strategy and study selection process, our team conducted multiple calibration exercises among reviewers before the formal screening process began. These calibration steps ensured a shared understanding of inclusion and exclusion criteria, reducing the likelihood of discrepancies during the screening phase. Given this iterative alignment, we did not find it necessary to apply additional agreement measures such as Cohen’s kappa coefficient. However, we acknowledge the importance of such metrics and have clarified our methodological approach in the manuscript.

We sincerely appreciate the reviewers' constructive feedback, which has helped us strengthen our manuscript. We hope that our revisions adequately address all concerns and that the manuscript is now suitable for publication in PLOS ONE.

Thank you for your time and consideration. We look forward to your response.

Best regards,

Dr Carla Trapé

University of São Paulo, Brazil
